# Inactivation of ATM/ATR DNA Damage Checkpoint Promotes Androgen Induced Chromosomal Instability in Prostate Epithelial Cells

**DOI:** 10.1371/journal.pone.0051108

**Published:** 2012-12-18

**Authors:** Yung-Tuen Chiu, Ji Liu, Kaidun Tang, Yong-Chuan Wong, Kum Kum Khanna, Ming-Tat Ling

**Affiliations:** 1 Department of Anatomy, The University of Hong Kong, Hong Kong, Special Administrative Region, China; 2 Department of Pathology, Faculty of Medicine, The University of Hong Kong, Hong Kong, Special Administrative Region, China; 3 Institute of Health and Biomedical Innovation, Queensland University of Technology, Queensland, Australia; 4 Signal Transduction Laboratory, Queensland Institute of Medical Research, Brisbane, Queensland, Australia; Wayne State University School of Medicine, United States of America

## Abstract

The ATM/ATR DNA damage checkpoint functions in the maintenance of genetic stability and some missense variants of the ATM gene have been shown to confer a moderate increased risk of prostate cancer. However, whether inactivation of this checkpoint contributes directly to prostate specific cancer predisposition is still unknown. Here, we show that exposure of non-malignant prostate epithelial cells (HPr-1AR) to androgen led to activation of the ATM/ATR DNA damage response and induction of cellular senescence. Notably, knockdown of the ATM gene expression in HPr-1AR cells can promote androgen-induced *TMPRSS2*: *ERG* rearrangement, a prostate-specific chromosome translocation frequently found in prostate cancer cells. Intriguingly, unlike the non-malignant prostate epithelial cells, the ATM/ATR DNA damage checkpoint appears to be defective in prostate cancer cells, since androgen treatment only induced a partial activation of the DNA damage response. This mechanism appears to preserve androgen induced autophosphorylation of ATM and phosphorylation of H2AX, lesion processing and repair pathway yet restrain ATM/CHK1/CHK2 and p53 signaling pathway. Our findings demonstrate that ATM/ATR inactivation is a crucial step in promoting androgen-induced genomic instability and prostate carcinogenesis.

## Introduction

Prostate cancer is currently the most commonly diagnosed male cancer in Western countries. When the cancer reaches the metastatic stage, the only frontline treatment available is androgen ablation therapy. Unfortunately, more than 70% of the patients will experience recurrence due to the development of a hormone-refractory stage. Currently, there is no effective second-line treatment available for patients at this stage. Therefore, it is important to understand the mechanisms responsible for prostate carcinogenesis and to identify a better prognostic marker for prostate cancer patients.

Similar to other cancers, genetic instabilities such as chromosome translocation are frequently detected in prostate cancer cells and are believed to play an initiating role in disease development. One of the prostate cancer-specific chromosome translocation is the fusion of *TMPRSS2* and *ERG* genes, which has been reported in 60–70% of prostate cancer tissues [1]. Other commonly detected translocations include fusion of TMPRSS2: ETV genes [2] and SLC45A3: ELK4 [3]. Although how these fusion genes may contribute to prostate carcinogenesis are still largely unknown, fusion transcripts such as TMPRSS2: ERG has been shown to drive prostate neoplastic development. The chromosomal rearrangement also occurs as an early event and continues to be expressed in metastatic and castration-resistant disease [2], suggesting that these products may at least be involved in disease progression.

Recently the male sex hormone androgen has been demonstrated to promote the recruitment of androgen receptor (AR) and topoisomerase II beta (TOP2B) to genomic breakpoints induced at androgen responsive genes including *TMPRSS2*: *ERG* fusion, the most common fusion detected in prostate cancer [4]. In a separate study, liganded-AR was also found to recruit endonuclease and deaminase at juxtaposed translocation loci and promote site-specific DNA double-stranded break [5]. More importantly, both studies demonstrated that transient androgen treatment resulted in induction of *TMPRSS2*: *ERG* fusion in prostate cancer cells, suggesting that androgen may play an important role in prostate cancer predisposition. In a more recent study, prolong androgen treatment was found to induce *TMPRSS2*: *ERG* fusion in the non-malignant prostate epithelial cells [6]. Interestingly, in the study by Lin et al, a transient androgen treatment was unable to induce *TMPRSS2*: *ERG* fusion in non-malignant prostate epithelial cells even in the presence of genotoxic stress, indicating the presence of repair mechanism in non-malignant prostate epithelial that suppress genetic instability, which has been abrogated in prostate cancer cells [5].

Genetic instabilities such as chromosome translocation trigger the activation of the ATM/ATR DNA damage checkpoint to arrest cell cycle and facilitate DNA repair [7], [8]. ATM is mainly activated by DNA double-strand breaks (DSBs) [9], while ATR responds to replication stress, although it is now recognized that the ATM pathway can also activate downstream components of the ATR arm following induction of DSBs in S-and G2 phases of cell cycle [10], [11]. Once activated ATM/ATR phosphorylate downstream effector proteins to initiate cell cycle checkpoints, and facilitate DNA repair through phosphorylating a number of its downstream targets such as checkpoint kinase 1 (Chk1), checkpoint kinase 2 (Chk2) and histone H2AX [12], [13], [14]. Interestingly, ATM has been reported to be highly activated in prostatic intraneoplasia (PIN), which is regarded as a precursor of prostate cancer [15]. Furthermore, some missense variants of the ATM gene have been shown to confer a moderate increased risk of prostate cancer. These observations suggest that the ATM DNA damage checkpoint acts as a barrier to initiation of prostate cancer, possibly through detecting and repairing the genetic instability that occurs during early stage of cancer development. Nonetheless, whether inactivation of this checkpoint plays a direct role in prostate cancer predisposition is still unknown.

In the present study, we provide evidence for the first time that androgen-induced activation of the ATM DNA damage checkpoint as well as the induction of cellular senescence in non-tumorigenic prostate epithelial cell (HPr-1 AR). More importantly, in the presence of androgen, inactivation of the ATM DNA damage checkpoint led to the induction of TMPRSS2/ERG fusion transcript in HPr-1 AR cells. Despite the fact that androgen treatment also induced ATM phosphorylation in prostate cancer cells (LNCaP), we were unable to detect any changes in the phosphorylation level of Chk1/2 or H2AX proteins, suggesting that the ATM DNA damage checkpoint can only be partially activated in prostate cancer cells. These results suggested that the ATM/ATR DNA damage checkpoint may play a crucial role in suppressing androgen-induced chromosome translocation in prostate epithelial cells, and inactivation of this checkpoint may facilitate androgen-induced genetic instability and prostate carcinogenesis.

## Results

### Androgen Activates ATM/ATR DNA Damage Checkpoint in HPr-1 AR Cells

**Figure 1:**
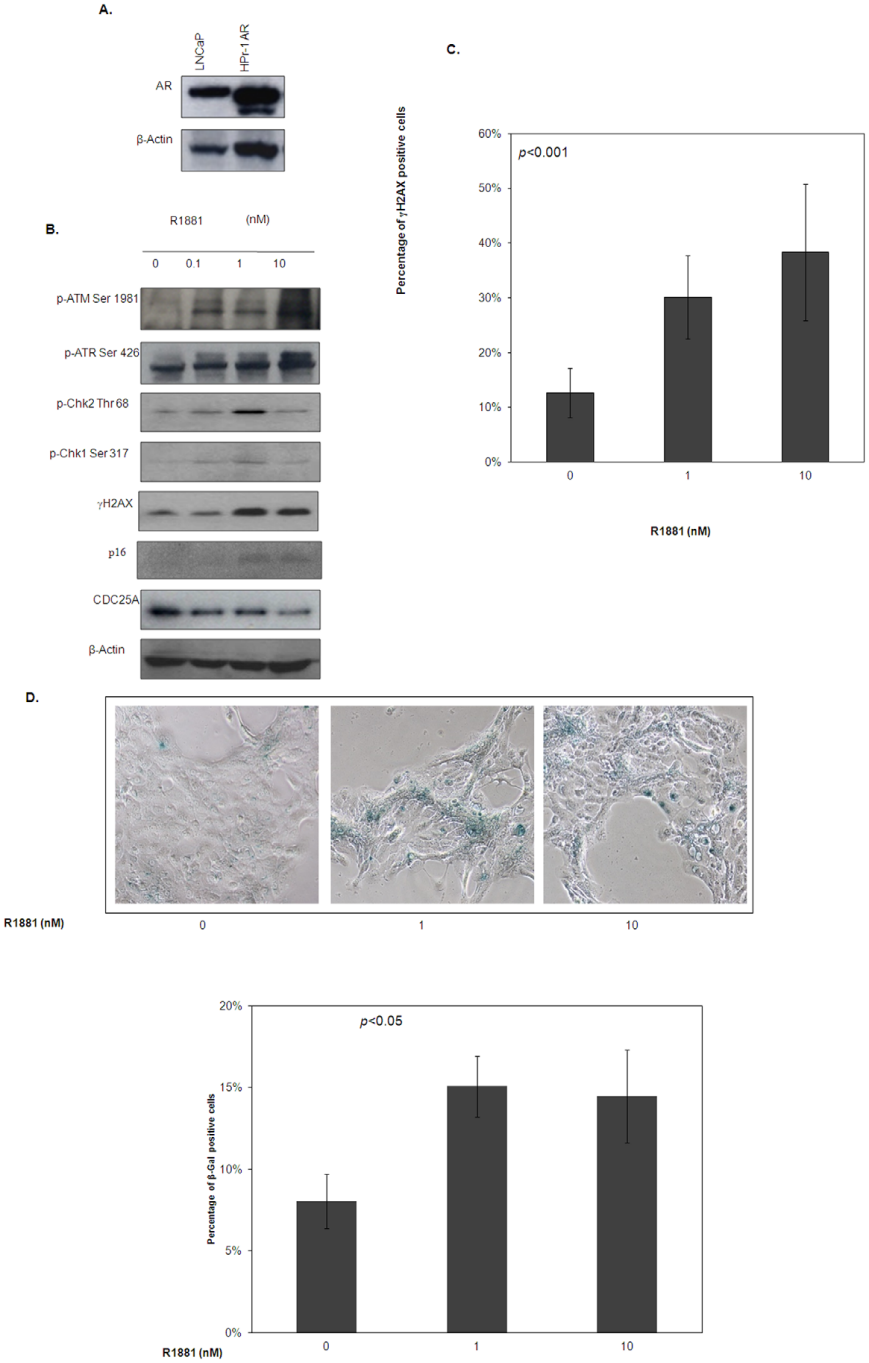
Effect of androgen on the activation of ATM/ATR DNA damage checkpoint in HPr-1 AR cells. (A) Expression of AR in HPr-1 cells were compared to that in LNCaP cells by Western blotting. (B) Androgen activates ATM/ATR DNA damage checkpoint in HPr-1 AR cells. Levels of phosphor-ATM (Ser 1981), phosphor-ATR (Ser 428), phosphor-Chk1 (Ser 317), phosphor-Chk2 (Thr 68), γH2AX and p16 in HPr-1 AR cells after 24 hours of R1881 treatment. (C) Androgen induces γH2AX foci formation in HPr-1AR cells. γH2AX foci was detected with immunofluorescent staining and counted under microscope. Result was presented as percentage of γH2AX positive cells. (D) Androgen induces cellular senescence in HPr-1 AR cell. Cells were treated with different dosages of R1881 for 6 days and stained with senescence-associated β-galactosidase for 16 hours. The images were captured under 200× magnification. The percentage of positively stained cells was calculated. The standard deviation of the means was used as error bars. *p*<0.05 was considered statistically significant as determined by student-*t* test.

Androgen induces prostate cancer-specific translocations of *TMPRSS2: ERG* in prostate cancer cells but not in non-malignant prostate epithelial cells [5]. We hypothesize that this may due to the activation of the ATM/ATR DNA damage checkpoint in the non-malignant cells, which may help in suppressing the androgen-induced chromosome instability. To test this hypothesis, an immortalized non-malignant prostate epithelial cell line was used as a model. The HPr-1 cells were first stably transfected with AR by using the lentiviral gene delivery system. As shown in Figure 1A, the AR protein expression level in the HPr-1 AR is comparable to that in LNCaP cells. The HPr-1 AR cells were then exposed to synthetic androgen analog R1881 for 24 hours, and the expression and phosphorylation levels of the DNA damage checkpoint proteins were determined. As shown in Figure 1B, phosphorylation level of ATM (Ser 1981) and ATR (Ser 426) was upregulated after R1881 treatment, demonstrating the activation of both ATM and ATR by androgen treatment. Phosphorylations of ATM/ATR downstream targets such as Chk1 (Ser 317) and Chk2 (Thr 68) were also observed upon androgen treatment. More importantly, the level of γ-H2AX, a sensitive and well-known DNA damage marker, was also increased after the treatment. This was further confirmed by immunofluorescence staining revealing an increase in the percentage of cells displaying >10 γ-H2AX foci in androgen-treated compared to non-treated HPr-1 AR cells (Figure 1C). These findings suggest that androgen treatment may induce DNA damage in non-malignant prostate epithelial cells, which led to activation of the ATM-ATR DNA damage checkpoint pathway.

ATM/ATR DNA damage checkpoint activation has previously been shown to induce cellular senescence, a major protective mechanisms against genetic instability [16]. Meanwhile, androgen treatment was also found to induce the expression of the senescence marker p16 (Fig. 1B). To investigate if androgen-induced ATM/ATR activation also triggers cellular senescence, HPr-1 AR cells were treated with R1881 or vehicle for 6 days and stained for senescence associated β-galactosidase (β-gal). As shown in Figure 1D, the percentage of β-gal positive cells (appear as blue-green) was significantly induced by R1881 treatment, indicating that HPr-1 AR cells undergo cellular senescence when exposed to androgen treatment.

### Knockdown of ATM Promotes Androgen-induced Chromosome Translocation in HPr-1 AR Cells

**Figure 2:**
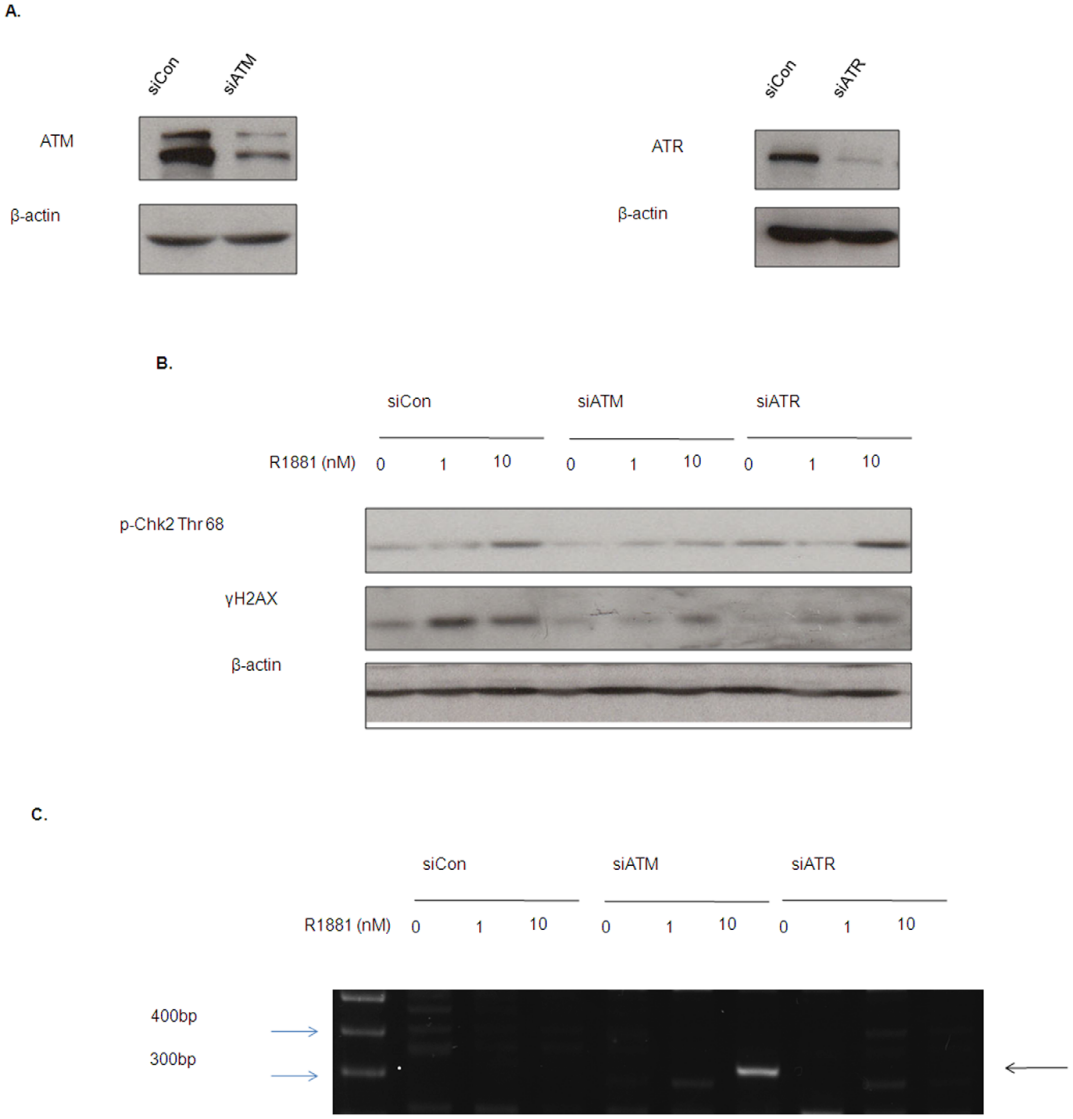
Knockdown of ATM/ATR promotes androgen-induced chromosome translocation in HPr-1 AR cells. (A) Knockdown of the ATM and ATR expression in HPr-1 AR cells. Cells were transiently transfected with scramble control siRNA (siCon), siATM and siATR for 48 hours and were harvested for Western blotting analysis. B) Androgen-induced γH2AX was suppressed in ATM/ATR deficient HPr-1 AR cells. Cells were transiently transfected with siCon, siATM and siATR and exposed to R1881 for 24 hours. Level of γH2AX was examined by Western blotting. C) Androgen induces chromosome translocation of *TMPRSS2: ERG* in ATM deficient HPr-1 AR cells. Cells were transiently transfected with scramble control siRNA (siCon), siRNA targeting ATM (siATM) and that targeting ATR (siATR) and treated with/without R1881 for 24 hours and harvested for RNA extraction. cDNA was then synthesized and the mRNA level of *TMPRSS2:ERG* fusion gene was analyzed by nested PCR. Note that *TMPRSS2: ERG* gene fusion transcript can only be detected in ATM-deficient HPr-1 AR cells that treated with androgen.

Next, we asked if inactivation of the ATM/ATR DNA damage checkpoint may facilitate androgen-induced *TMPRSS2: ERG* fusion. We then knockdown the expression of either the ATM or ATR gene in HPr-1 AR cells by transiently transfecting the cells with ATM siRNA (siATM) or ATR siRNA (siATR). As shown in Figure 2A, transfection of siATM and siATR effectively knockdown levels of ATM and ATR protein, respectively, in HPr-1 AR cells as compared to the scramble control (siCon). Examination of γH2AX expression revealed that knockdown of ATM or ATR both suppressed the induction of γH2AX by androgen treatment (Figure 2B), suggesting that the androgen-induced DNA damage response was significantly suppressed by ATM/ATR knockdown. Consistent with the previous findings [4], [5], short-term treatment of the non-malignant prostate epithelial cells (HPr-1 AR) with androgen did not induce *TMPRSS2: ERG* fusion transcript (Figure 2C).More importantly, we were able to detect a *TMPRSS2: ERG* fusion transcript (Figure 2C) in the ATM-deficient HPr-1 AR cells treated with androgen. However, transient knockdown of ATR was able to induce the same fusion transcript, confirming that the ATM DNA damage checkpoint is acting as a surveillance system to guard against the androgen-induced chromosome translocation.

### Androgen Induces Partial Activation of the ATM DNA Damage Checkpoint in LNCaP Cells

**Figure 3:**
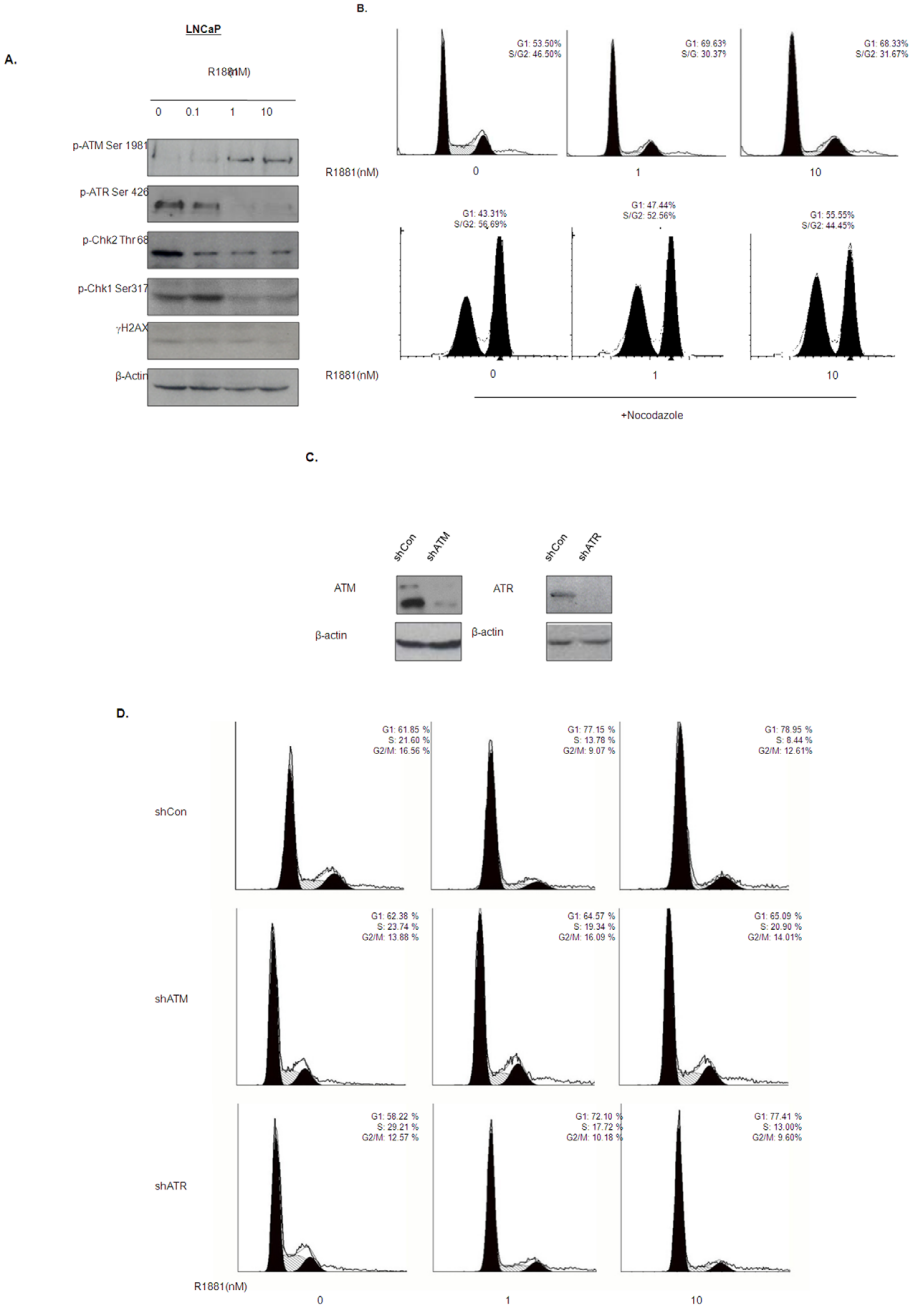
Effect of androgen on activation of ATM/ATR DNA damage checkpoint in LNCaP cells. (A) Levels of phosphor-ATM (Ser 1981), phosphor-ATR (Ser 428), phosphor-Chk2 (Thr 68), phosphor-Chk1 (Ser317) and γH2AX in LNCaP cells after 24 hrs of R1881 treatment. (B) Androgen induces G1 arrest in LNCaP cells. LNCaP cells supplemented with R1881 alone (upper panel) or together with nocodazole (lower panel) for 24 hours were harvested and fixed for cell cycle analysis using flow cytometry. The percentages represent cell population at different phases of the cell cycle. (C) Protein expression of ATM and ATR in LNCaP-shCon, shATM and shATR transfectants were examined by Western blotting. (D) Androgen fails to induce G1 arrest in shATM transfectants. shCon, shATM and shATR transfectants were treated with different doses of R1881 for 24 hours and cells were fixed for flow cytometry analysis. Table showed the percentage change of G1 phase and S phase LNCaP cells after supplemented with 1 nM R1881 for 24 hours. Androgen fails to suppress cell proliferation in shATM LNcaP transfectant.

The fact that androgen treatment alone can induce *TMPRSS2: ERG* fusion in the prostate cancer, LNCaP, cell line suggests that these cells may contain a detective ATM/ATR DNA damage checkpoint. We therefore tested if androgen exposed LNCaP cells also activates the same DNA damage response pathway as reported above for HPr-1 AR (non-malignant prostate epithelial) cells. Phosphorylation levels of the DNA damage checkpoint proteins were examined in LNCaP cells after 24 hours of androgen (R1881) treatment. Similar to HPr-1 AR cells, ATM phosphorylation level was significantly increased when LNCaP cells were exposed to R1881 (Figure 3A). Notably, LNCaP cells showed constitutive phosphorylation of ATR (Ser 428), Chk2 (Thr 68) and Chk1 (Ser 317) and these were all decreased after the androgen (R1881) treatment. Meanwhile, γH2AX level remained unchanged during the treatment. These results suggested that androgen-induced DNA damage response pathway is partially impaired in LNCaP cells.

Although androgen treatment did not induce the activation of the ATM/ATR downstream proteins, we were still able to detect the G1 cell cycle arrest of LNCaP cells after the treatment (Figure 3B). To investigate if the cell cycle arrest is the consequence of the partial activation of the ATM/ATR DNA damage checkpoint, stable LNCaP sublines with ATM (shATM) and ATR (shATR) knockdown were generated by lentiviral gene delivery system. Western blotting results in Figure 3C showed that the ATM and ATR were effectively knockdown in shATM and shATR transfectants when compared to the control (shCon) transfectants. We next analyzed the effect of androgen treatment (R1881) on cell cycle profile of these transfectants. Similar to the parental cells, shCon or shATR transfected LNCaP-cells underwent G1 arrest after treatment with R1881 (Figure 3D). Moreover, the treatment did also result in suppression of the number of viable cells in both LNCaP-shCon and shATR transfectants (Figure S1). However, ATM-deficient LNCaP (LNCaP-shATM) cells failed to significantly alter the percentage of cells in G1 phase of cell cycle after R1881 treatment (Figure 3D). Consistent with the cell cycle analysis, R1881 also failed to affect the viable cell number in ATM-deficient LNCaP cells (Figure S1). These findings illustrate that androgen induces G1 cell cycle arrest through an ATM-dependent and ATR-independent mechanism.

### Differential Regulation of the ATM/ATR Downstream Targets in LNCaP cells after Androgen Treatment

**Figure 4:**
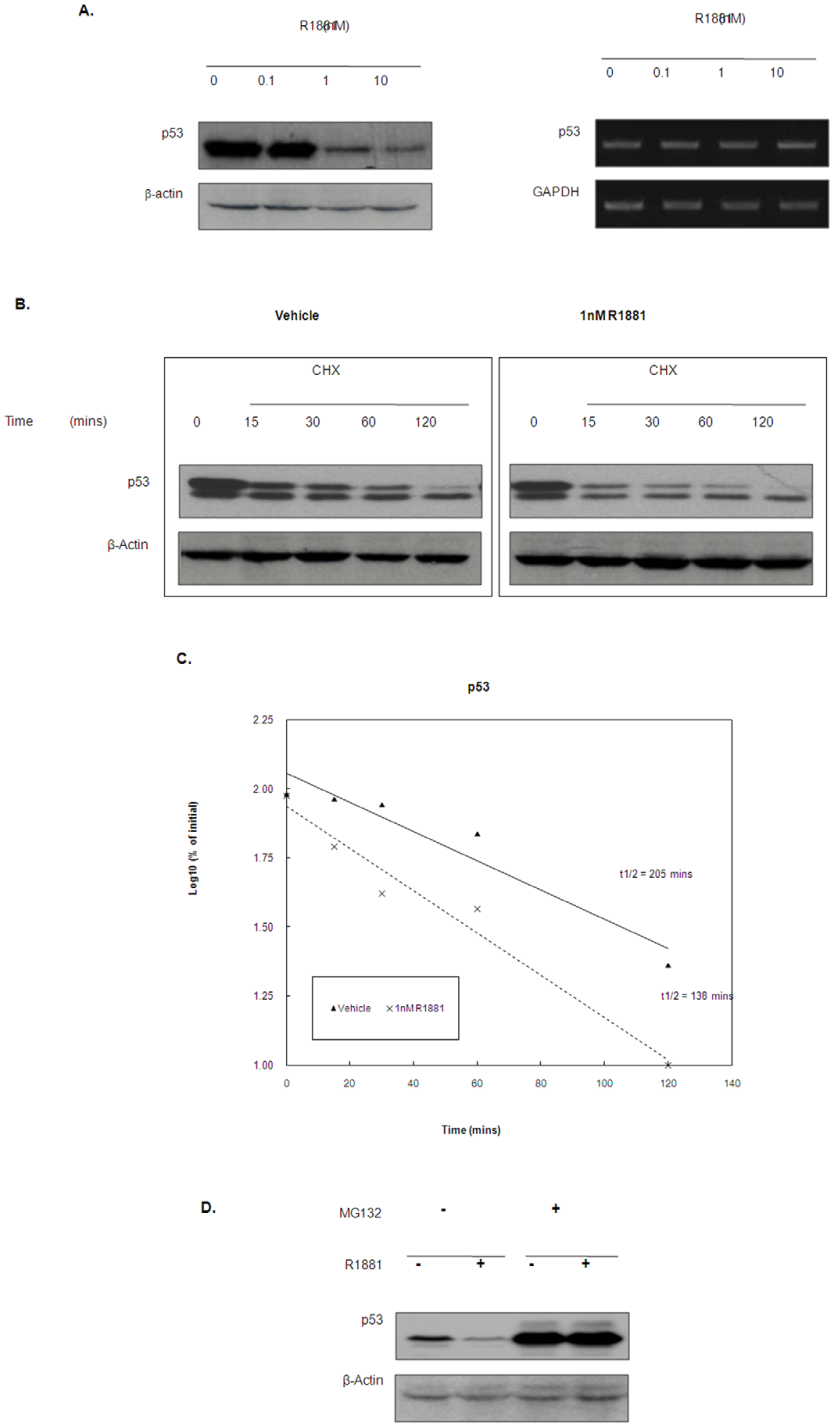
Differential effect of androgen on the regulation of ATM/ATR downstream targets in LNCaP cells. (A) Androgen down regulates p53 protein expression in LNCaP cells. LNCaP cells were treated with R1881 for 24 hours and harvested for Western blotting analysis on p53 protein expression (Left panel). p53 mRNA levels in androgen-treated LNCaP cells were examined by RT-PCR and GAPDH expression level was used as a loading control (Right panel). (B) Androgen promotes p53 protein degradation in LNCaP cells. Degradation profile of p53 protein in LNCaP cells with or without R1881 (1 nM) treatment was examined by blocking protein synthesis with CHX (50 µg/ml). p53 protein level was measured at the indicated time points by Western blotting. Signal intensity of the Western blotting result was measured by gel documentation system and the reading was normalized as percentage to that of the initial p53 level (level at time = 0). (C) Log_10_ of the percentage was plotted against time and the half-life of the p53 protein was calculated as the time corresponding to the log_10_ of 50%. (D) Androgen fails to down regulate p53 in the presence of proteasome inhibitor. LNCaP cells were treated with 1 nM R1881 for 24 hrs. At 16 hrs of R1881 treatment, 2 µM of the proteasome inhibitor (MG132) was added. At the end of the treatment, cells were lysed for western blotting analysis using p53 antibody.

Given that master regulators of the G1 arrest are p53 and CDC25A, two of the major downstream effectors of the ATM/ATR DNA damage checkpoint, this prompted us to examine the regulation of this pathway in LNCaP cells. p53 is phosphorylated by Chk1/2 in response to DNA damage, which leads to its stabilization [17], [18], [19]. Consistent with the decrease in phosphorylation of Chk1 and Chk2, p53 protein level was also found to be downregulated by androgen treatment in LNCaP cells (Figure 4A). The fact that p53 mRNA level remain constant after the treatment while degradation of p53 protein was accelerated (Figure 4C) indicated that the downregulation of p53 by androgen is due to destabilization of the protein, possibly due to the decrease in Chk1/2 activity. This is further confirmed by treatment of LNCaP cells with the proteasome inhibitor (MG132), which completely abolished the effect of androgen on p53 proteins (Figure 4D).

**Figure 5:**
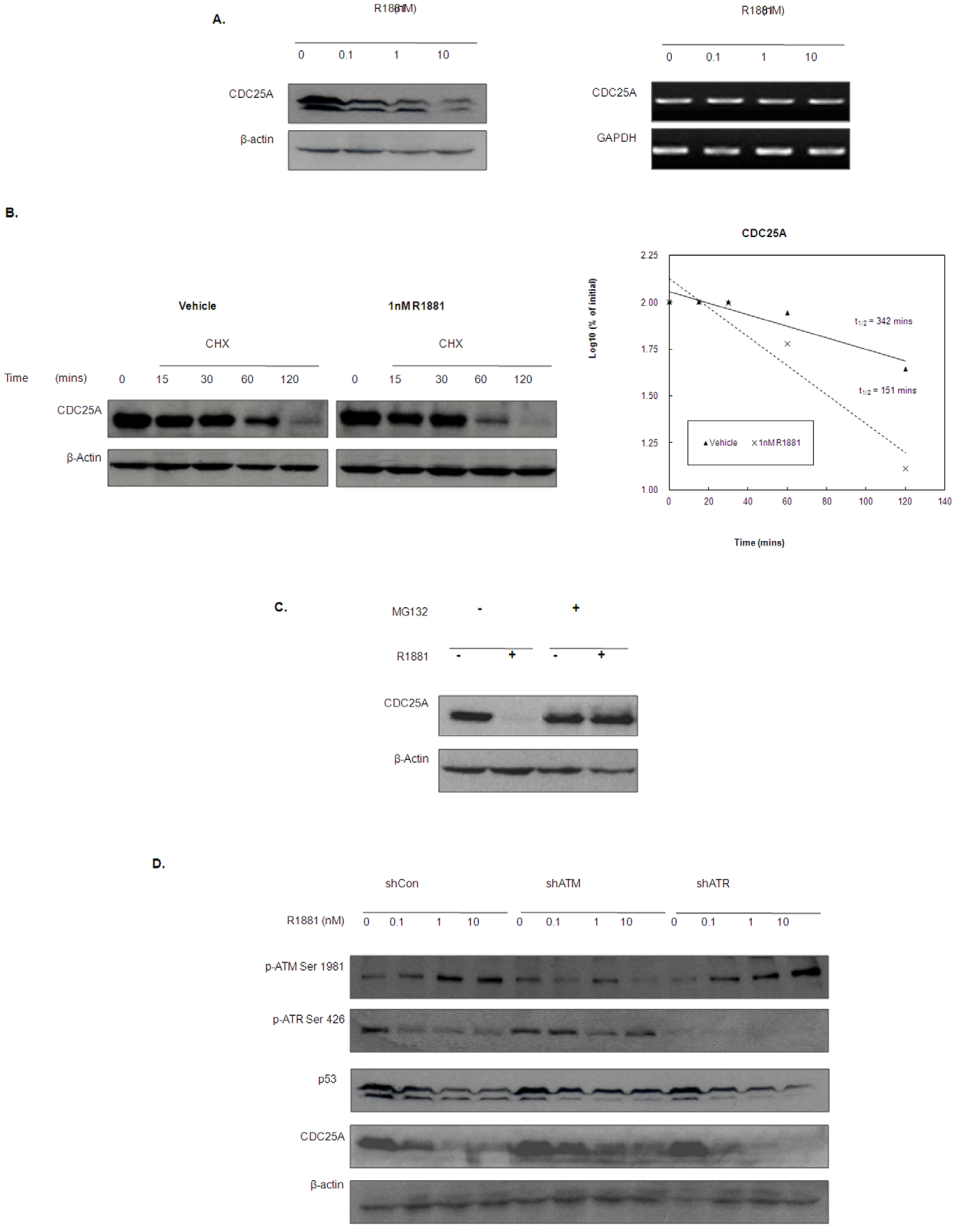
Androgen downregulates CDC25A protein in an ATM dependent manner. (A) LNCaP cells were treated with R1881 for 24 hours and harvested for Western blotting analysis and RT-PCR on CDC25A protein and mRNA expression. β-actin (WB) and GAPDH (RT-PCR) were used as a loading control. (B) Androgen promotes CDC25A protein degradation in LNCaP cells. Degradation profile of CDC25A protein in LNCaP cells with or without R1881 (1 nM) treatment was examined by blocking protein synthesis with CHX (50 µg/ml). CDC25A protein level was measured at the indicated time points by Western blotting. Signal intensity of the Western blotting result was measured by gel documentation system and the reading was normalized as percentage to that of the initial CDC25A level (level at time = 0). Log_10_ of the percentage was plotted against time and the half-life of the CDC25A protein was calculated as the time corresponding to the log_10_ of 50% (right panel). (C) Androgen fails to down regulate CDC25A in the presence of proteasome inhibitor. LNCaP cells were treated with 1 nM R1881 for 24 hrs. At 16 hrs of R1881 treatment, 2 µM of the proteasome inhibitor (MG132) was added. At the end of the treatment, cells were lysed for Western blotting analysis using CDC25A antibody. (D) Knockdown of ATM, but not ATR, partially abolishes the effect of androgen on CDC25A expression. shCon, shATM and shATR transfectants were treated with different doses of R1881 for 24 hours and were lysed for Western blotting analysis.

Next, we examined the effect of androgen on CDC25A, which is phosphorylated by Chk1/2 in response to DNA damage [20], [21]. Unlike p53, phosphorylation of CDC25A is known to destabilize the protein, leading to induction of cell cycle arrest. Intriguingly, R1881 was found to downregulate CDC25A in a dose dependent manner (Figure 5A), even though the phosphorylation levels of Chk1/2, indicative of activation status, were suppressed by the treatment, suggesting that androgen downregulates CDC25A in a Chk1/2 independent manner. Meanwhile, both CDC25A mRNA and promoter activity were not affected by R1881 treatment (Figure 5A & Figure S2), suggesting that the decrease in protein expression is mediated through post-transcriptional mechanism. This is confirmed by determining the degradation profiles of CDC25A protein in androgen-treated and -untreated LNCaP cells (Figure 5B). Moreover, treatment of LNCaP cells with the proteasome inhibitor (MG132) completely abolished the effect of androgen on CDC25A proteins (Figure 5C). These results further suggest that androgen downregulates CDC25A through a proteasome-mediated protein degradation pathway.

To investigate if androgen regulates p53 and CDC25A protein levels through the ATM/ATR DNA damage checkpoint, we tested the effect of androgen on p53 and CDC25A expression in shATM and shATR transfectants when compared to the control (shCon). As shown in Figure 5D, knockdown of ATM, but not ATR, partially recovered the CDC25A protein expression in LNCaP cells, suggesting that downregulation of CDC25A by androgen requires the activation of ATM. Consistent with these findings even the transient knockdown of ATM (siATM), but not ATR in LNCaP cells completely abolished the effect of androgen on CDC25A protein expression (Figure S3). However, neither stable nor transient knockdown of ATM or ATR abolish the effect of androgen on p53 level. These results suggested that androgen destabilized CDC25A, but not p53 protein through activating the ATM-dependent DNA damage response pathway that leads to G1 arrest in prostate cancer cells.

## Discussion

In the present study, we reported that androgen activates the ATM/ATR DNA damage response and induces cellular senescence in non-malignant prostate epithelial cells. Furthermore, inactivation of ATM/ATR led to accumulation of the androgen-induced chromosome translocation. Our results demonstrate for the first time the cooperative effect of androgen and DNA damage response inactivation in prostate cancer predisposition.

Androgen has recently been shown to induce prostate specific chromosomal translocation in LNCaP cells concomitantly treated with genotoxic stress. Intriguingly, the same treatment was unable to induce any detectable chromosomal translocation in non-malignant prostate epithelial cells [5], although a prolonged exposure to androgen was found to induce the TMPRSS2/ERG fusion transcript [6]. A possible reason for this disparity could be the differences in the integrity of the DNA damage response between normal and cancer cells. In fact, in our study the treatment of HPr-1AR cells with androgen were found to result in activation of both ATM and ATR, leading to the phosphorylation of Chk1/2 and the induction of γH2AX (Figure 1A). However, in LNCaP cells, the same treatment only induced the phosphorylation of ATM (Figure 3A) without activation of downstream targets suggesting that these cells may have a defective androgen-induced activation of DNA damage response. In fact, we observed constitutive phosphorylation of ATR and CHK1 and CHK2 which was substantially decreased upon exposure to androgen. The failure of androgen to induce γH2AX in LNCaP cells (Figure 3A) further highlighted the presence of defective androgen-induced DNA damage response in prostate cancer cells.

More importantly, we showed that non-malignant prostate epithelial cells (HPr-1AR) become susceptible to androgen-induced chromosomal translocation after transient knockdown of ATM (Figure 2C), further demonstrating the crucial role of the ATM DNA damage response in the maintenance of chromosome stability in non-malignant cells. Indeed, some missense variants of ATM gene mutation have previously been shown to confer increased risk of prostate cancer [22], [23]. In the study by Angele et al, one out of the five ATM variants (P1054R) was found to associate with increased risk of prostate cancer development [22]. In another study, the same ATM variant was also reported to cause a twofold increase in the risk of developing prostate cancer [23]. Thus, our findings may help to understand the association between ATM mutation and prostate cancer development.

It is interesting to note that HPr-1AR cells treated with androgen undergo cellular senescence eventually (Figure 1D). Cellular senescence, a process of irreversible arrest of cell division, is one of the safeguard mechanisms to prevent cancer formation. Previous reports have shown that senescence can be induced by DNA damage in tumor cells through an ATM/ATR dependent mechanism [24], [25], [26]. In addition, cells entering senescence display increased γH2AX foci [25], [27], [28]. The induction of cellular senescence and γH2AX foci in HPr-1 AR cells by androgen treatment suggests that androgen may induce DNA damage, leading to activation of ATM/ATR DNA damage response. Indeed, by inactivating the ATM/ATR DNA damage response pathway, we were able to mimic the response of LNCaP cells to androgen treatment in HPr-1AR cells, as evidenced by the induction of TMPRSS2/ERG fusion transcript and the lack of γH2AX induction after androgen treatment (Figure 2B & C). These results indicate the potential defects in mounting optimal response to androgen-induced DNA damage in prostate cancer cells, which may in-turn explain the escape of prostate cancer cells from cellular senescence.

Unlike HPr-1AR cells, LNCaP cells treated with androgen was found to undergo cell cycle arrest (Figure 3B), which is consistent with previous study [29]. However, we found that the G1 arrest induced by androgen is in fact associated with degradation of the tyrosine phosphatase CDC25A (Figure 5A–C), which is one of the cell cycle proteins regulated by the ATM-dependent DNA damage response [30], [31]. Consistent with this, knockdown of ATM not only recovered the CDC25A protein level, but also completely abolished the effect of androgen on G1 arrest (Figure 3D and Figure 5D). In regards to ATM functioning to regulate CDC25A levels in LNCaP cells treated with androgen, the mechanism is unlikely to be Chk1/2 dependent given that phosphorylation of both is decreased after androgen exposure (Figure 3A). However, it is noteworthy that, recently, Raf-1/ERK pathway, activated in LNCaP by androgen treatment [32], was found to phosphorylate CDC25A at the Chk1/2 phosphorylation site and to induce its degradation [33]. Since Raf-1/ERK has been shown to be required for ATM DNA damage checkpoint functioning [34], it is thus possible that in the absence of Chk1/2 activation, androgen exposure induces ATM mediated CDC25A degradation through Raf-1/ERK activation.

In summary, we have demonstrated the effect of androgen on the activation of ATM/ATR DNA damage response and the consequent induction of senescence in non-malignant prostate epithelial cells. Notably, this pathway is partially impaired in prostate cancer cells. Collectively, these findings establish that inactivation of ATM pathway is a crucial step in promoting androgen-induced *TMPSS2: ERG* chromosome translocation and the consequent genomic instability and prostate carcinogenesis. Considering the role of androgen in the pathology of prostate cancer, our findings may provide a possible linkage between androgen, genomic instability and prostate carcinogenesis.

## Materials and Methods

### Cell Culture

Human prostate cancer cell line LNCaP was obtained from American Type Culture Collection (Rockville, MD). Prostate epithelial cell line HPr-1 was was described in the previous study [35]. LNCaP was maintained in medium RPMI 1640 (Invitrogen, Carlsbad, CA) supplemented with 2% penicillin-streptomycin (P/S) (Invitrogen, Carlsbad, CA) and 5% fetal bovine serum (FBS) (Invitrogen, Carlsbad, CA). HPr-1 was maintained in keratinocyte-serum free medium (Invitrogen, Carlsbad, CA) supplemented with 1% P/S. All cell types were kept at 37°C, 5% CO_2_. For experiments, the cells were incubated in RPMI medium supplemented with 5% (v/v) charcoal-dextran-treated fetal bovine serum (CSFBS) for 24 hrs before androgen supplementation. The synthetic androgen methyltrienolone (R1881) (Perkin-Elmzer, Waltham, MA) was dissolved in absolute ethanol at a concentration of 100 mM. The proteasome inhibitor, MG132, and cycloheximide (CHX) (Calbiochem, San Diego, CA) were dissolved in DMSO at concentration of 10 mM and 100 mg/ml respectively.

### siRNAs Transient Transfection

The siGENOME non-targeting siRNA pool #1 (siCon), ON-TARGET plus SMARTpool siRNA human ATR (siATR) and ON-TARGET plus SMARTpool siRNA human ATM (siATM) were purchased from Dharmacon, Chicago, IL. They were transfected into the cells using Lipofectamine™ 2000 reagent (Invitrogen, Carlsbad, CA) following the manufacturer’s instruction. Twenty-four hours after transfection, cells were either lysed for western blotting analysis or treated with R1881 for 72 hr before lysed for mRNA extraction and RT-PCR analysis.

### Generation of Stable Knockdown Transfectants

The HPr-1 AR overexpressing transfectants (HPr-1 AR) was generated by using pLenti6-AR expression vector. LNCaP ATM (LNCaP shATMi) and ATR (LNCaP shATRi) knockdown transfectants were generated by using pLKO.1 ATM shRNA and ATR shRNA expression vectors respectively. The Mission™ non-target shRNA control vector SHC002 (Sigma, St. Louis, MO) was used for the generation of the corresponding control. Lentivirus were generated and used for infecting HPr-1 and LNCaP cells with protocol described in our previous studies [36].

### Western Blotting

Western blotting was carried out as described previously [37]. The antibodies were purchased from following suppliers: CDC25A, AR, p16 and β-actin (Santa Cruz, CA, USA); Phospho-ATM (Ser1981), Phospho-ATR (Ser 428), Phospho-Chk1 (Ser317), Phospho-Chk2 (Thr68), ATM and ATR (Cell Signaling Technology Inc); phospho- Histone H2A.X (Ser 139) (Millipore Corporation, Billerica, MA); p53 (Dakocytomation, Glostrup, Denmark).

### Dual Luciferase Reporter Assay

pGL3-CDC25A-Luc was a gift from Professor Daniel DiMaio (Yale University School of Medicine, New Haven, Connecticut, U.S.). LNCaP cells were plated in 12-wells plates at 20% confluency and were transfected with pGL3-CDC25A-Luc and the internal control pRL-TK-Luc. The transfectants were then treated with methyltnenolone (R1881) for another 24 hrs. Cells were then lysed and assayed for luciferase activity using Dual-Luciferase® Reporter Assay System (Promega, Madison, WI) following manufacturer’s instruction. The pRL-TK-Luc was used as an internal control and the experiment was performed in triplicate.

### 3-[4, 5-Dimethylthiazol-2-yl]-2, 5-diphenyltetrazolium Bromide (MTT) Assay

Cells were grown in 96-well plate with 100 µl culture medium. At indicated time point, 10 µl of MTT labeling reagent (5 mg/ml, in PBS) was added to each wells and incubated for 4 hours at 37°C. After 4-hour incubation, the formazan crystals formed were dissolved with 200 µl of DMSO (Sigma, St. Louis, MO) and incubated for 5 minutes at 37°C. After that, absorbance at 570 nm was measured using Labsystem multiscan microplate reader (Merck Eurolab, Dietikon, Schweiz). 100 µl culture medium was set as blank control and experiment was performed in triplicate.

### Immunofluorescent Staining

Cells were seeded on 8-well chamber slide and fixed with 4% paraformaldehyde in phosphate-buffered saline (PBS) and washed once with PBS. The cells were then permeablilized with 1% Triton X-100 in PBS. Cells were blocked with 1% BSA in PBS for 20 minutes and incubated with anti-phospho-Histone H2A.X (Ser139) (γ-H2AX) antibody (1∶200, Milipore, Billerica, MA) for 2 hours at room temperature. Cell were washed thrice with PBS and incubated with relevant fluorescein-labeled secondary antibody. Cellular DNA content was counterstained with 0.5 µg/ml 4′ 6-diamidino-2-phenylindole (DAPI). Fluorescent signals were visualized with Carl Zeiss Imager Z1 microscope with Apotome slider (Carl Zeiss, Inc., Thornwood, NY) and photographed using Axiovision software. At least 250 cells were counted under 200× magnification for each experiment and cells containing >10 fluorescent foci was counted as positively stained. The standard deviation of the means for at least three independent experiment was used as error bars. *P*<0.05 was considered statistically significant as determined by student-*t* test.

### Senescence-associated β-galatocidase Staining (SA β-gal)

Cells were seeded on 12-well plate at 20% confluency. After 6 days treatment, cells were washed once with 500 µl 1×PBS (pH 6.0) and fixed with 1× fixative solution (2% formaldehyde and 0.2% glutaraldehyde in PBS). After the fixation, cells were then washed with 1×PBS twice and stained with the Senescence-associated β-galatocidase staining kit (Cell signaling, Beverly, MA). Positive cells were counted under microscope. At least 500 cells were counted from 3 random fields and the percentage of positively stained cells was calculated. The standard deviation of the means was used as error bars. *P*<0.05 was considered statistically significant as determined by student-*t* test.

### Reverse Transcriptase- polymerase Chain Reaction (RT-PCR)

Total RNA was isolated using TRIZOL® reagent (Invitrogen, Carlsbad, CA) following the manufacturer’s instructions. Two micrograms of cDNA was synthesized by using SuperScript™ First-Strand Synthesis System for RT-PCR (Invitrogen, Carlsbad, CA) and PCR was carried out with GeneAmp® PCR System 9700 (Applied Biosystems, Foster City, CA). The CDC25A primers sequences are 5′-GCC TCT CGT GGC AGG GCA GTC-3′ and 5′-CAT CAC CTG GCC TGA GGA ATC-3′; p53 primers sequences are 5′-TCA GAT CCT AGC GTC GAG CCC-3′ and 5′-GGG TGT GGA ATC AAC CCA CAG-3′; GAPDH primers sequences are 5′-ACC ACA GTC CAT GCC ATC AC-3′ and 5′-TCC ACC ACC CTG TTG CTG TA-3′. Glyceraldehyde-3-phosphate dehydrogenase (GAPDH) expression was examined as an internal control.

TMPRSS2-ERG fusion product was detected with a two-step nested PCR. First PCR was performed with primers described by Lin et al [5], while the nested PCR was performed with the primers described by Clark et al [38] with expected size of ∼300 bp. PCR products were resolved in agarose gel and the image was captured with a gel documentation system.

### Cell Cycle Analysis

Cells were plated in 5% FBS culture medium at 20% confluency and incubated in 5% CSFBS culture medium for 24 hours before R1881 treatment. Where indicated, nocodazole was added into the medium 8 hrs after the addition of R1881. The cells were then harvested and fixed in ice cold 70% ethanol. The cells were then washed with phosphate-buffered saline (PBS) before incubation with propidium iodide (50 µg/ml) and RNase (1 µg/ml) for 30 mins. Cell cycle analysis was performed on a flow cytometer EPICS profile analyzer and analyzed using the ModFit LT2.0 software (Coulter, Miami, FL).

### Determination of Half-life of Proteins

The procedures for determination of protein half-life were as described previously [39]. Cells were treated with 1 nM R1881 and collected after treatment with protein synthesis inhibitor cycloheximide (50 ug/ml) for the indicated time point. Cells were then lysed for Western blotting with anti-CDC25A and anti-p53 antibodies. The band intensity of the Western blotting result was measured by gel documentation system with the reading normalized as percentage of the initial CDC25A level (level at time = 0). The percentages of CDC25A and p53 level were then plotted against time in Log scale. Slope was calculated from the plot and was used to generate the half-life (t = 1/2) of the CDC25A and p53 protein, which is the time required for degradation of 50% of the initial protein.

## Supporting Information

Figure S1 **shCon, shATM and shATR transfectants were treated with different doses of R1881 for 5 days and MTT assay was performed.** The experiment was performed in triplicates and the mean and standard deviation were calculated.
(TIF)

Figure S2 **CDC25A promoter activity was determined in androgen-treated LNCaP cells by luciferase reporter assay.** TK promoter activity was used as the internal control.
(TIF)

Figure S3 **LNCaP cells were transient transfected with non-targeting siRNA (siCon) and siRNA targeting ATM (siATM).** Cells were then treated with R1881 for 24 hours and then harvested for Western blotting analysis.
(TIF)
