# Supplementary figures and images for: Inactivation of ATM/ATR DNA Damage Checkpoint Promotes Androgen Induced Chromosomal Instability in Prostate Epithelial Cells
Source: PLoS One. 2012 Dec 18;7(12):e51108. doi: 10.1371/journal.pone.0051108 (PMC3525593; doi:10.1371/journal.pone.0051108)

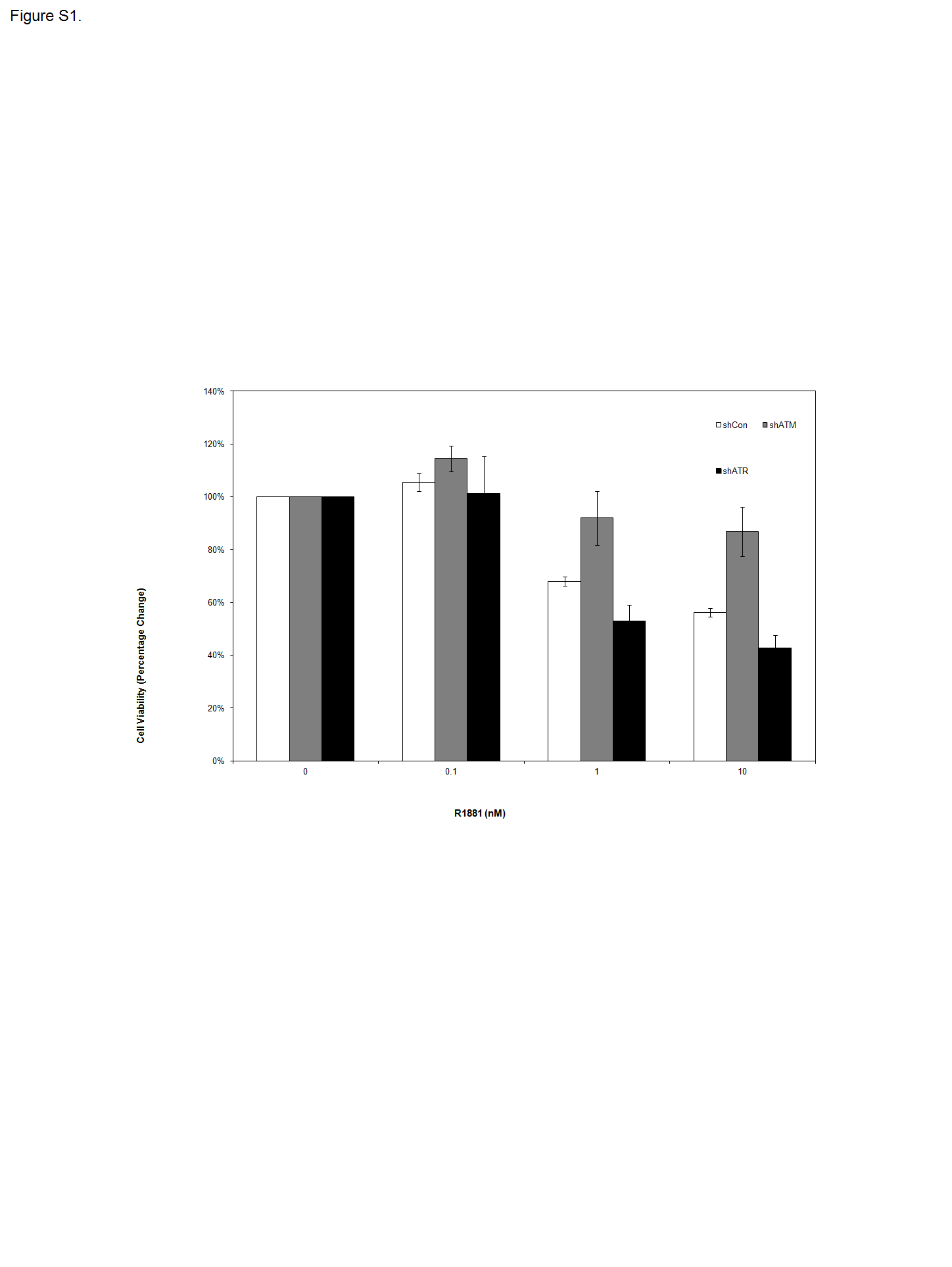

Supplement: Figure S1 — shCon, shATM and shATR transfectants were treated with different doses of R1881 for 5 days and MTT assay was performed. The experiment was performed in triplicates and the mean and standard deviation were calculated. (TIF) [file pone.0051108.s001.tif]

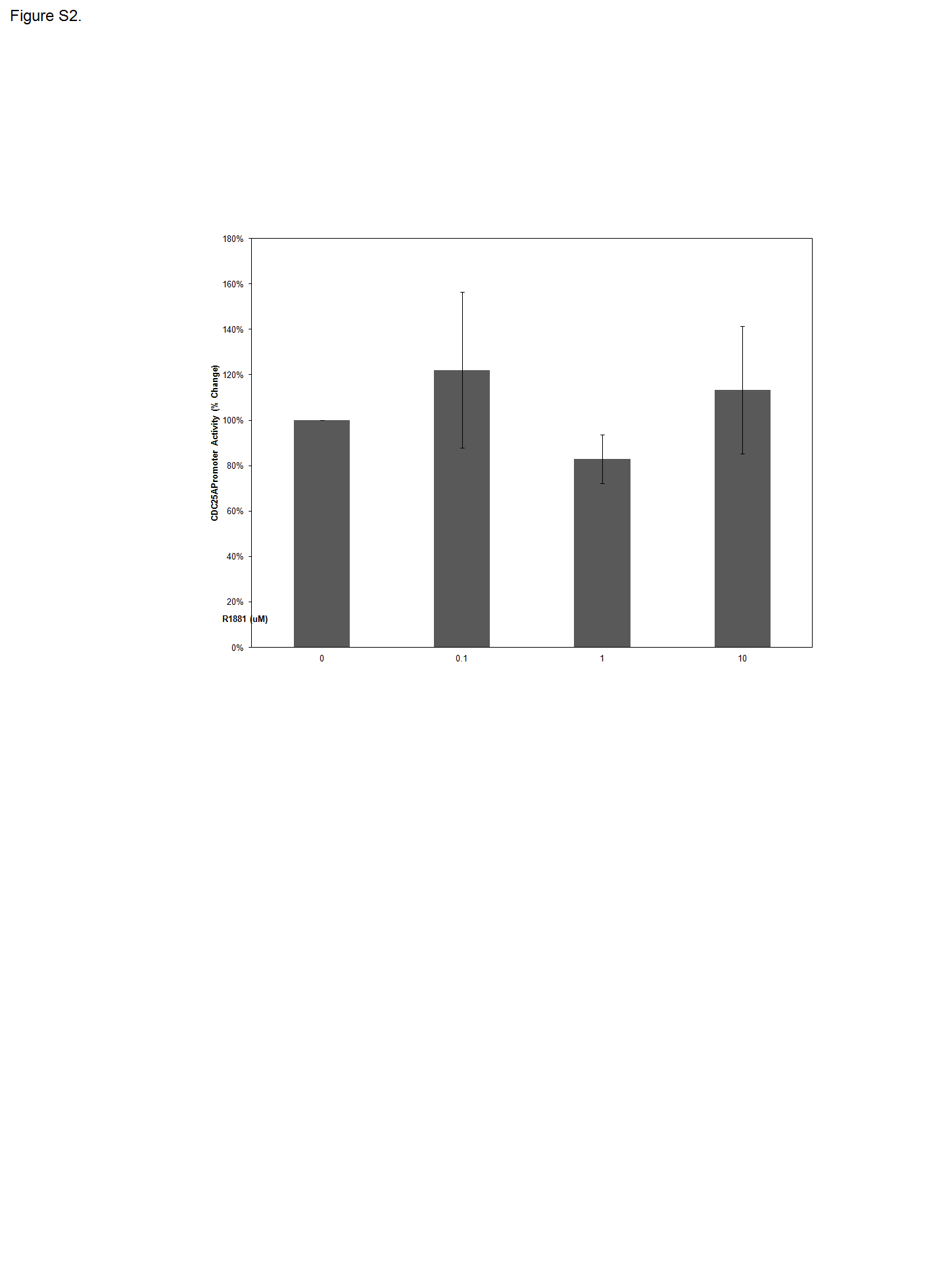

Supplement: Figure S2 — CDC25A promoter activity was determined in androgen-treated LNCaP cells by luciferase reporter assay. TK promoter activity was used as the internal control. (TIF) [file pone.0051108.s002.tif]

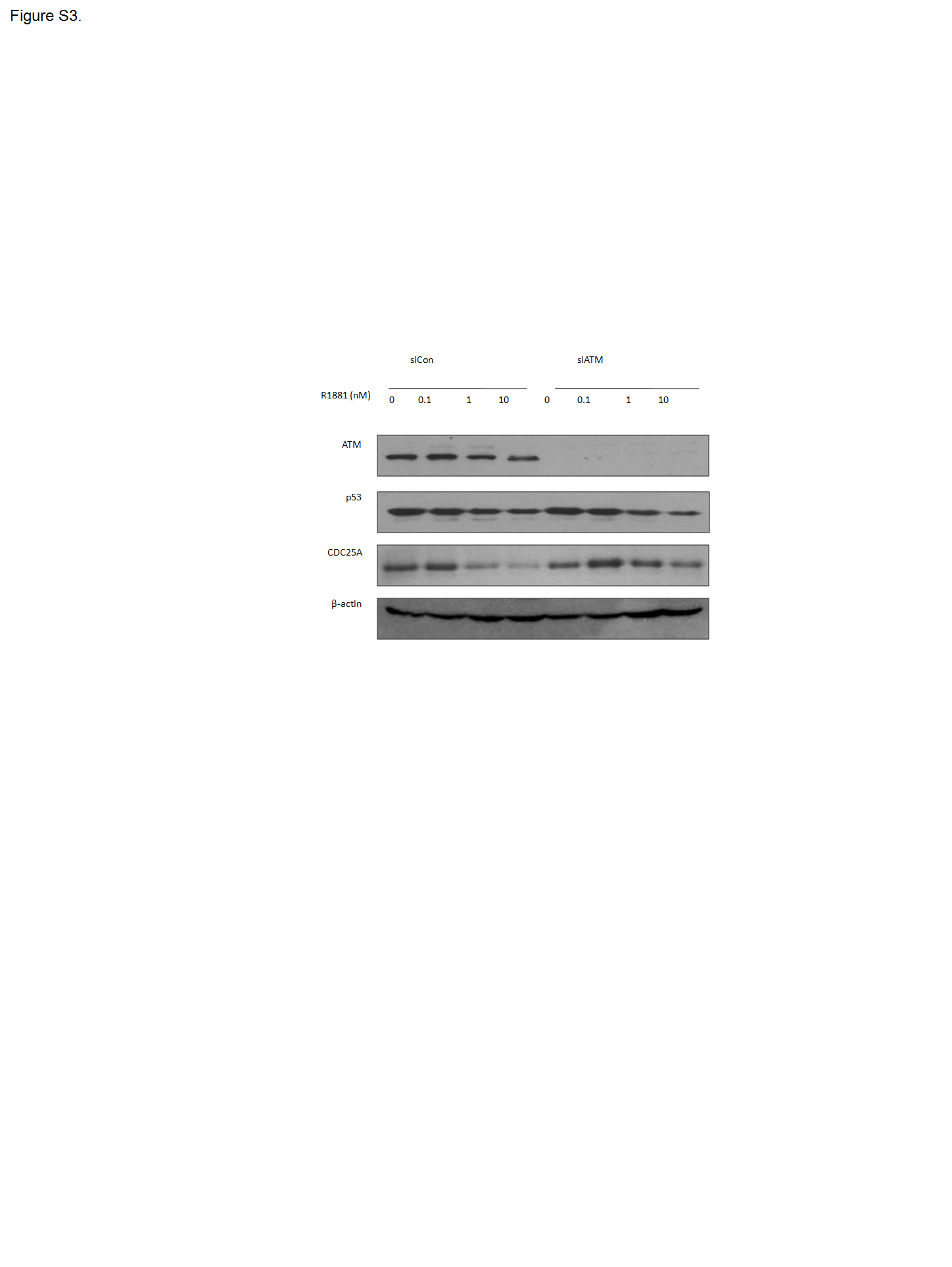

Supplement: Figure S3 — LNCaP cells were transient transfected with non-targeting siRNA (siCon) and siRNA targeting ATM (siATM). Cells were then treated with R1881 for 24 hours and then harvested for Western blotting analysis. (TIF) [file pone.0051108.s003.tif]
